# Is Academic Self-efficacy a Protective Factor for the Well-Being of Female Freshmen from an Underrepresented Population?

**DOI:** 10.1192/j.eurpsy.2026.11955

**Published:** 2026-07-31

**Authors:** S. Aamir, M. Bo Julaia, M. Pilotti

**Affiliations:** Core Humanities & Social Sciences, Prince Mohammad Bin Fahd University, Al Khobar, Saudi Arabia

## Abstract

**Introduction:**

Undergraduate students at the start of their academic journey are vulnerable to diminished well-being due to increased academic demands and other changes to their everyday routines. Most research related to the well-being of freshmen has been devoted to populations of the Global North. Particularly neglected are Saudi Arabian female students who have been recently given agency rights. Female freshmen are expected not only to adapt to the demands of academic life but also to counteract archaic gender stereotypes, as well as to succeed in fields that are still dominated by men. While they are navigating uncharted territories, their well-being is largely unknown.

**Objectives:**

This research surveyed a sample of young female freshmen from Saudi Arabia. Three dimensions of diminished well-being were examined: stress, anxiety, and depression. It was hypothesized that freshmen would mostly report suffering from stress since stress is a response to real or perceived challenges, such as those presented by novel academic demands (H1).

In student populations (e.g., Ampuero-Tello et al. 2022), academic self-efficacy (beliefs about one’s ability to achieve desired academic outcomes) is often linked to lower stress, anxiety, and depression. Thus, our study asked whether academic self-efficacy could be treated as a protective factor for Saudi Arabian female students’ well-being. We expected academic self-efficacy to be inversely related to stress, anxiety, and depression (H2).

**Methods:**

Participants were 200 Saudi Arabian female freshmen enrolled at an English-medium institution (age range: 18-28 years). After offering informed consent, students completed DASS-21 (Antony et al., 1998), which measured depression, anxiety, and stress. Academic self-efficacy was measured through an adaptation of the questionnaire developed by Chen et al. (2001), which asked students to consider their academic life before answering each statement of general confidence in their abilities. Cronbach’s Alpha ranged from 0.78 to 0.88.

**Results:**

Table 1 illustrates the percentage of students by DASS-21 levels. In the table, the severe and extremely severe levels were combined. The average academic self-efficacy was 18.97 (*SD* = 3.97; range = 0-32). Table 2 displays Pearson correlations with significant values in bold.

The incidence of severe anxiety was high compared to other symptoms. H1 was not supported. However, self-efficacy was linked to a lower incidence of all symptoms of distress, thereby supporting H2.

**Image 1:**
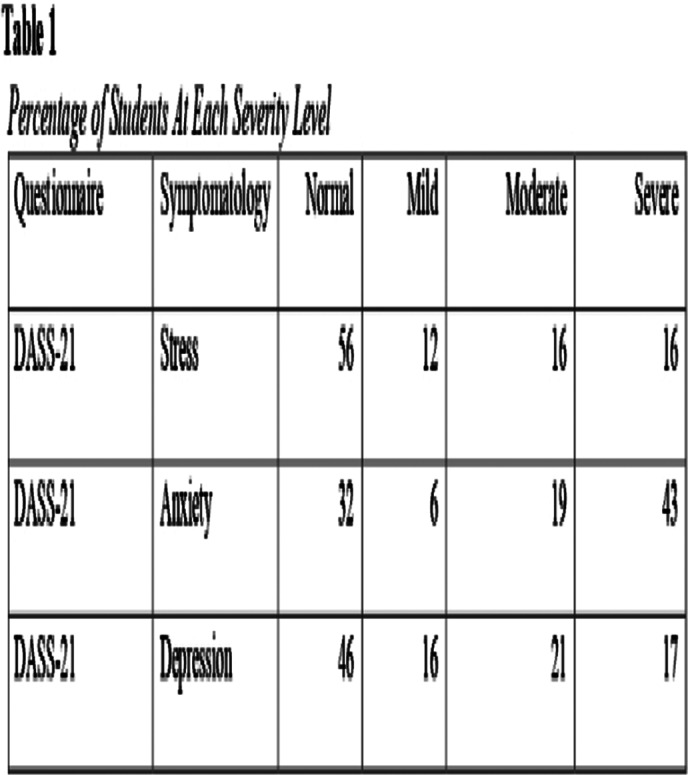

**Image 2:**
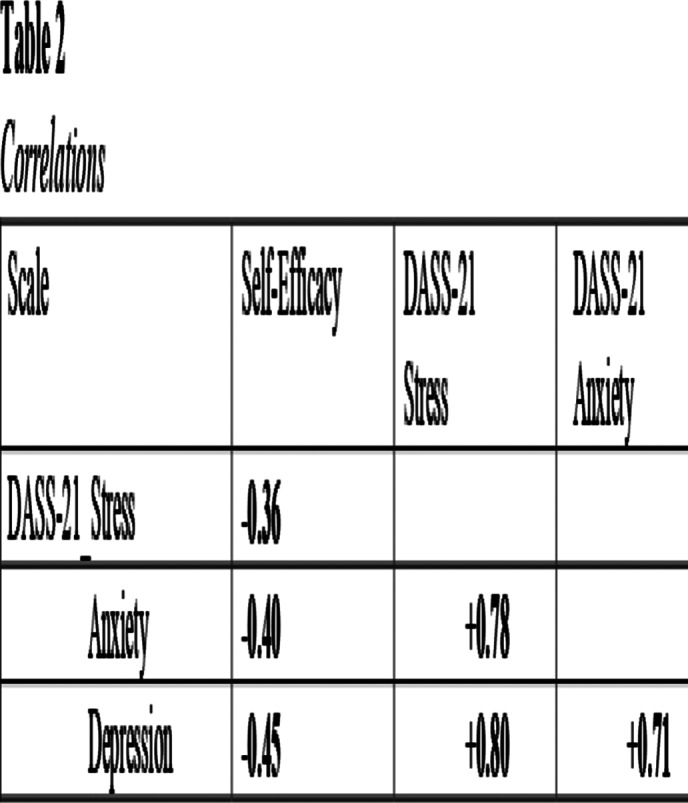

**Conclusions:**

Although ours is a correlational study, its results suggest that the freshman year may be accompanied by disproportionate levels of anxiety. As such, targeted interventions, focusing on self-efficacy, are needed to ensure the well-being of this student population.

**Disclosure of Interest:**

S. Aamir Employee of: None., M. Bo Julaia Employee of: None., M. Pilotti Employee of: None.

